# Supplementary material for: The interactome of intact mitochondria by cross-linking mass spectrometry provides evidence for coexisting respiratory supercomplexes
Source: Mol Cell Proteomics. 2017 Dec 8;17(2):216–32. doi: 10.1074/mcp.RA117.000470 (PMC5795388; doi:10.1074/mcp.RA117.000470)
Supplement: Supplemental Data [file supp_RA117.000470_133922_0_supp_23978_fzffwf.zip › spectra_annotation/mito_spectra_annotation/384-1-3-1-6-1.pdf]

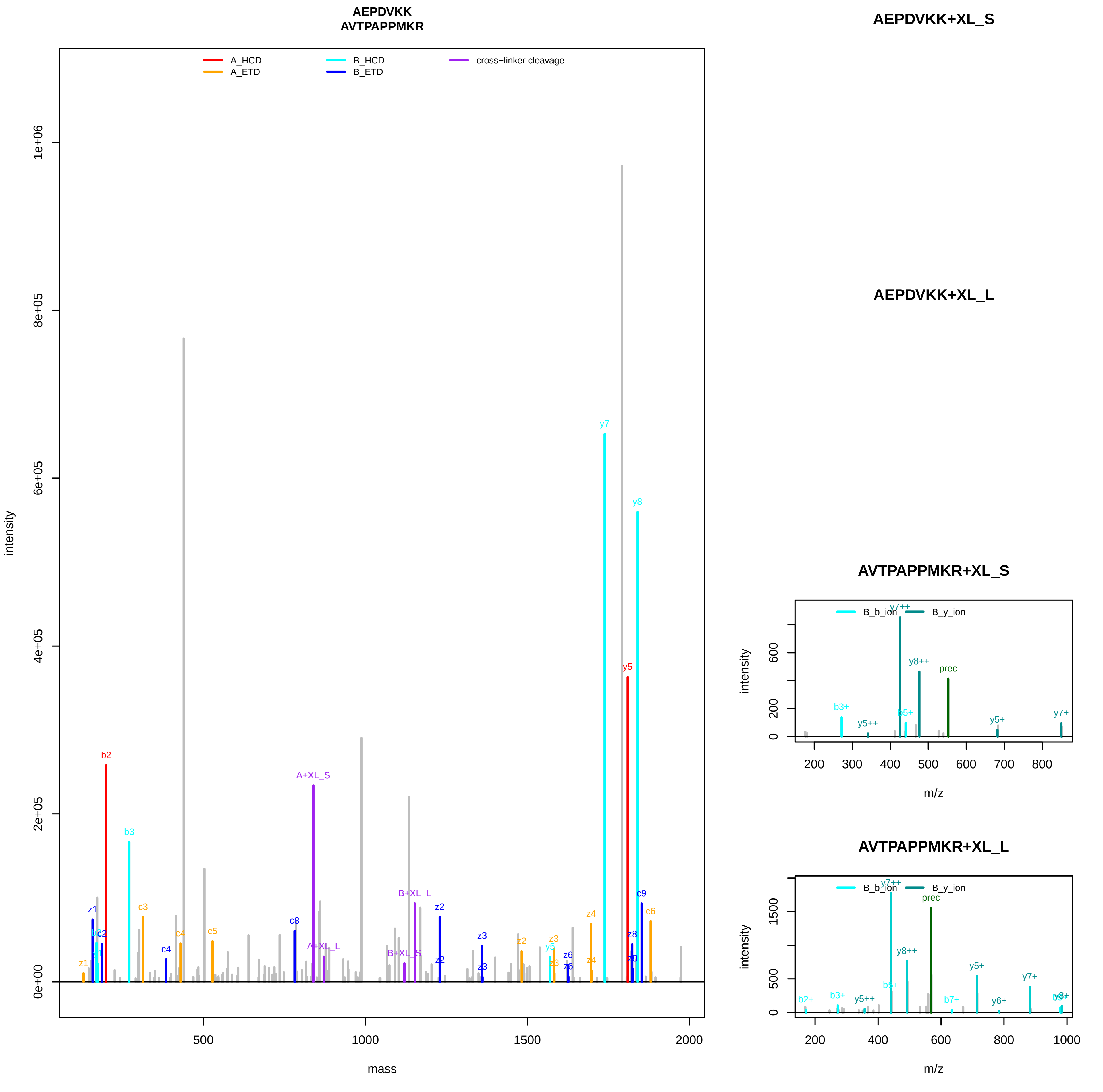
AEPDVKK
AVTPAPPMKR
A_HCD
A_ETD
B_HCD
B_ETD
cross−linker cleavage
intensity
mass
0e+00
2e+05
4e+05
6e+05
8e+05
1e+06
500
1000
1500
2000
b2
b3
c3
c4
c5
c6
c8
c9
z1
z2
z3
z4
z6
z8
y5
y7
y8
A+XL_S
A+XL_L
B+XL_L
B+XL_S
AEPDVKK+XL_S
AEPDVKK+XL_L
AVTPAPPMKR+XL_S
AVTPAPPMKR+XL_L
B_b_ion
B_y_ion
m/z
prec
b3+
b5+
b2+
b7+
b8+
y5++
y7++
y8++
y5+
y6+
y7+
y8+
0
200
600
500
1500
100
300
400
700
800
1000
